# A validation study of the Occupational Depression Inventory in Poland and Ukraine

**DOI:** 10.1038/s41598-024-54995-w

**Published:** 2024-02-22

**Authors:** Krystyna Golonka, Karine O. Malysheva, Dominika Fortuna, Bożena Gulla, Serhii Lytvyn, Leon T. De Beer, Irvin Sam Schonfeld, Renzo Bianchi

**Affiliations:** 1 Institute of Applied Psychology, Jagiellonian University, Kraków, Poland; 2 Faculty of Psychology, Taras Shevchenko National University of Kyiv, Kyiv, Ukraine; 3 Doctoral School of Social Sciences, Jagiellonian University, Kraków, Poland; 4 Department of Psychology, Norwegian University of Science and Technology (NTNU), Trondheim, Norway; 5 WorkWell Research Unit, North-West University, Potchefstroom, South Africa; 6 Department of Psychology, The City College of the City University of New York, New York City, NY USA; 7 Department of Psychology, Norwegian University of Science and Technology (NTNU), Dragvoll, Building 12, Level 5, 7491 Trondheim, Norway

**Keywords:** Job-related distress, Factor analysis, Mokken scale analysis, Occupational health, Burnout, Psychometrics, Psychology, Medical research

## Abstract

This study examined the psychometric and structural properties of the Polish and Ukrainian versions of the Occupational Depression Inventory (ODI). We relied on two samples of Polish employees (*N*_Sample1_ = 526, 47% female; *N*_Sample2_ = 164, 64% female) and one sample of Ukrainian employees (*N*_Sample3_ = 372, 73% female). In all samples, the ODI exhibited essential unidimensionality and high total-score reliability (e.g., McDonald’s omegas > 0.90). The homogeneity of the scale was strong (e.g., 0.59 ≤ scale-level *H*s ≤ 0.68). The ODI’s total scores thus accurately ranked individuals on a latent occupational depression continuum. We found evidence of complete measurement invariance across our samples, a prerequisite for between-group comparisons involving observed scores. Looking into the criterion validity of the ODI, we found occupational depression to correlate, in the expected direction, with resilience and job-person fit in six areas of working life—workload, control, rewards, community, fairness, and values. The prevalence of occupational depression was estimated at 5% in Sample 1, 18% in Sample 2, and 3% in Sample 3. Our findings support the use of the ODI’s Polish and Ukrainian versions. This study adds to a growing corpus of research suggesting that the ODI is a robust instrument.

## Introduction

Job-related distress is a pressing concern within the field of occupational health science due to its detrimental effects on individuals’ well-being, health, and longevity^1–4^. The Occupational Depression Inventory (ODI) was recently developed^5–7^ to respond to challenges in how job-related distress has been conceptualized and measured. The instrument has garnered growing attention from occupational health specialists since its introduction^8^. The ODI is designed to evaluate depressive symptoms specifically ascribed to work-related experiences. In its development, the instrument drew upon the nine core diagnostic symptoms for major depression outlined in the *Diagnostic and Statistical Manual of Mental Disorders*, fifth edition (DSM-5)^9^. Unlike “traditional” depression scales, the items of the ODI incorporate causal attributions to work (e.g., “My experience at work made me feel like a failure”)^5–7^. Causal attributions have been widely employed in psychological and medical sciences to explore etiological pathways and establish diagnoses of stress-related disorders, including acute stress disorder and posttraumatic stress disorder^9^. Moreover, causal attributions have played a pivotal role in measuring various constructs in work and organizational psychology, such as work motivation^10^.

The ODI has undergone validation in multiple languages—e.g., English, French, Italian, Spanish, Swedish, Brazilian-Portuguese—and countries—e.g., the USA, France, Italy, Spain, Sweden, Brazil^5–7,11–17^. The instrument has consistently demonstrated robust psychometric and structural properties. Using advanced statistical techniques, such as exploratory structural equation modeling (ESEM) bifactor analysis, investigators have found the ODI to exhibit high factorial validity and to meet the requirements for essential unidimensionality. An essentially unidimensional scale is a scale that, while presenting a degree of multidimensionality, is sufficiently unidimensional to be used based on its total score (i.e., to be treated as a one-factor measure). Essential unidimensionality is particularly worthy of examination in the context of bifactor modeling. In addition, the ODI has displayed strong total-score reliability. Concerning criterion validity, the ODI has shown associations with multiple work-related and work-unrelated variables, including workplace violence, sick leave, economic stress, antidepressant usage, general health status, effort-reward and demand-control imbalances at work, objective cognitive performance, companies’ stock growth, and states’ economic deprivation^5,11–18^.

The present study inquired into the psychometric and structural properties of the Polish and Ukrainian versions of the ODI. More specifically, we focused on the factorial validity, dimensionality, homogeneity, total-score reliability, and cross-sample measurement invariance of the ODI. The study additionally offers a glimpse into the instrument’s criterion validity by investigating the association of occupational depression with resilience and job-person fit in six areas of working life—workload, control, rewards, community, fairness, and values. Because resilience and job-person fit are expected to promote well-being at work and successful coping with work-related stressors^19,20^, we hypothesized that occupational depression would be negatively associated with this set of variables. Among many reports, the STADA Health Report 2022, a large-scale survey of approximately 30,000 respondents from 15 countries, suggests that Europe may be on the brink of a mental health crisis, with the magnitude of job-related distress being particularly elevated in Eastern European countries^21^. Such findings underline the importance of making the ODI available in countries such as Poland and Ukraine. From a broader perspective, assessing job-related distress reliably and validly has been challenging and may benefit from democratizing access to innovative instruments such as the ODI^22–25^.

## Methods

### Study samples and recruitment procedures

The first sample (Sample 1) consisted of 526 Polish employees (47% female; *M*_AGE_ = 40, *SD*_AGE_ = 10, age range = 18–60). The sample was recruited through Biostat (https://www.biostat.com.pl/), an online consumer panel provider that is commonly used by researchers in Poland.

The second sample (Sample 2) comprised 164 Polish employees (64% female; *M*_AGE_ = 41, *SD*_AGE_ = 9, age range = 22–65). The sample was recruited through StrongUJ (https://stronguj.project.uj.edu.pl/), an online platform dedicated to employee support and career development^26^.

The third sample (Sample 3) consisted of 372 Ukrainian employees (73% female; *M*_AGE_ = 40, *SD*_AGE_ = 15, age range = 17–83). The sample was recruited through public announcements on various Facebook pages and Telegram channels, as well as among students from the Faculty of Psychology at the Taras Shevchenko National University of Kyiv. The eligibility criteria included being at least 18 years old, having proficiency in the Ukrainian language, and possessing some work experience.

Participation in the study was voluntary. All participants provided informed consent. The study was conducted in accordance with the ethical regulations of the host institutions and the principles of the Declaration of Helsinki. The part of the study pertaining to Poland complied with the demands of, and received approval from, the institutional review board of Jagiellonian University. The part of the study pertaining to Ukraine complied with the demands of, and received approval from, the institutional review board of the Taras Shevchenko National University of Kyiv.

### Measures of interest

#### ODI

The ODI comprises nine core symptom items (rated from 0 for “never or almost never” to 3 for “nearly every day”) and a supplementary question gauging turnover intention^5^. The nine core symptom items assess the nine diagnostic symptoms of major depressive disorder described in the DSM-5^9^. The symptoms are assessed within a two-week time window, consistent with the DSM-5. The ODI is intended to be used based on its total score (dimensional approach) and/or the diagnostic algorithm that accompanies the instrument (categorical approach). The diagnostic algorithm can be found in the form of an SPSS syntax in Supplemental Materials 1 and 2. The diagnostic algorithm is described in detail in the inaugural ODI paper^5^. The ODI was translated into Polish and Ukrainian using a back-translation method^27^. Native speakers first translated the original English version into Polish and Ukrainian. Then, different native speakers translated the Polish and Ukrainian versions back into English. We did not detect significant discrepancies between the original and back-translated English versions. The ODI’s Polish and Ukrainian versions are displayed in Table 1 and additionally provided together with instructions to respondents in Supplemental Materials 1 (for Poland) and 2 (for Ukraine). Descriptive statistics are available in Table 2. The prevalence of occupational depression was 5% in Sample 1, 18% in Sample 2, and 3% in Sample 3.

**Table 1:** Polish and Ukrainian versions of the occupational depression inventory (ODI).

Symptoms	Items
Anhedonia	Moja praca była tak stresująca, że nie mogłem/am cieszyć się rzeczami, które zwykle lubię robić Moя poбoтa бyлa нacтiльки нaпpyжeнoю, щo я нe мiг(мoглa) нacoлoджyвaтиcь тим, щo зaзвичaй люблю poбити [My work was so stressful that I could not enjoy the things that I usually like doing]
Depressed mood	Czułem/am się przygnębiony/a z powodu mojej pracy Я пoчyвaв(лa) ceбe пpигнiчeним(нoю) чepeз мoю poбoтy [I felt depressed because of my job]
Sleep alterations	Stres związany z pracą spowodował, że miałem/am problemy ze snem (miałem/am trudności z zasypianiem, wybudzałem/am się lub spałem/am znacznie więcej niż zwykle) Cтpec нa poбoтi пpизвiв дo пpoблeм зi cнoм (мeнi бyлo вaжкo зacнyти aбo пiдтpимyвaти coн, aбo я cпaв(лa) нaбaгaтo бiльшe, нiж зaзвичaй) [The stress of my job caused me to have sleep problems (I had difficulties falling asleep or staying asleep, or I slept much more than usual)]
Fatigue/loss of energy	Czułem/am się wyczerpany/a swoją pracą Я пoчyвaв(лa) ceбe виcнaжeним(нoю) чepeз мoю poбoтy [I felt exhausted because of my work]
Appetite alterations	Czułem/am, że stres w pracy wpłynął na mój apetyt (straciłem/am apetyt lub przeciwnie, jadłem/am za dużo) Я вiдчyвaв(лa), щo мiй aпeтит пopyшивcя чepeз cтpec нa poбoтi (я втpaтив(лa) aпeтит, aбo, нaвпaки, їв(їлa) зaнaдтo бaгaтo) [I felt my appetite was disturbed because of the stress of my job (I lost my appetite, or the opposite, I ate too much)]
Feelings of worthlessness	Moje doświadczenie w pracy sprawiają, że czuję się jak nieudacznik Te, щo я пepeживaв(лa) нa poбoтi, змycилo мeнe вiдчyвaти ceбe нeвдaxoю [My experience at work made me feel like a failure]
Cognitive impairment	Moja praca tak bardzo mnie stresowała, że miałem/am problem ze skupieniem się na wykonywanych czynnościach (np. na czytaniu artykułu w gazecie) lub z jasnym myśleniem (np. podejmowaniem decyzji) Moя poбoтa дyжe мeнe нaпpyжyвaлa, i я мaв(лa) пpoблeми з кoнцeнтpaцiєю нa тoмy, щo poблю (нaпpиклaд, читaння cтaттi), aбo з яcним миcлeнням (нaпpиклaд, пpийняття piшeння) [My job stressed me so much that I had trouble focusing on what I was doing (e.g., reading a newspaper article) or thinking clearly (e.g., to make decisions)]
Psychomotor alterations	W wyniku stresu w pracy czułem/am się niespokojny/a lub wręcz przeciwnie, czułem/am wyraźne spowolnienie—np. w sposobie poruszania się lub mówienia Унacлiдoк cтpecy нa poбoтi я вiдчyвaв(лa) ceбe нecпoкiйним(нoю), aбo, нaвпaки, пoмiтнo yпoвiльнeним(нoю)—нaпpиклaд, цe пpoявлялocь y тoмy, як я pyxaвcя(лacь) aбo гoвopив(лa) [As a result of job stress, I felt restless, or the opposite, noticeably slowed down―for example, in the way I moved or spoke]
Suicidal ideation	Pomyślałem/am, że wolałbym/abym umrzeć niż pozostać w tej pracy Я дyмaв(лa), щo кpaщe вмepти, нiж пpoдoвжyвaти пpaцювaти нa цiй poбoтi [I thought that I'd rather be dead than continue in this job]
Turnover intention (SQ)	Jeśli napotkałeś przynajmniej niektóre z wyżej wymienionych problemów, czy te problemy sprawiają, że zastanawiasz się nad odejściem z obecnej pracy lub stanowiska? Якщo ви cтикнyлиcя xoчa б з дeякими з пpoблeм, зaзнaчeниx вищe, чи цi пpoблeми пpивeли вac дo poзглядy питaння пpo звiльнeння з вaшoї пoтoчнoї poбoти aбo пocaди? [If you have encountered at least some of the problems mentioned above, do these problems lead you to consider leaving your current job or position?]

The full ODI form (including the instructions to respondents) is available in Polish in Supplemental Material 1 and in Ukrainian in Supplemental Material 2. Each file also includes an SPSS syntax implementing the provisional diagnosis algorithm of the ODI. SQ: subsidiary question.

**Table 2:** Descriptive statistics for the Occupational Depression Inventory.

Indicators	ODI1	ODI2	ODI3	ODI4	ODI5	ODI6	ODI7	ODI8	ODI9	Total score
Sample 1 (*N* = 526; Poland)										
Mean	0.86	0.88	0.82	1.13	0.71	0.63	0.71	0.72	0.46	0.77
Median	1.00	1.00	1.00	1.00	0.00	0.00	0.00	0.00	0.00	0.56
Mode	0	0	0	1	0	0	0	0	0	0.00
Standard deviation	0.92	0.90	0.94	0.96	0.92	0.88	0.90	0.90	0.80	0.73
Skewness (*SE* = 0.11)	0.87	0.78	0.83	0.46	1.08	1.22	1.06	1.12	1.62	0.91
Kurtosis (*SE* = 0.21)	− 0.10	− 0.22	− 0.43	− 0.74	0.10	0.45	0.10	0.34	1.54	0.04
Minimum	0	0	0	0	0	0	0	0	0	0.00
Maximum	3	3	3	3	3	3	3	3	3	3.00
Sample 2 (*N* = 164; Poland)										
Mean	1.50	1.87	1.46	2.11	1.41	1.22	1.30	1.21	0.48	1.40
Median	1.00	2.00	1.00	2.00	1.00	1.00	1.00	1.00	0.00	1.44
Mode	1	2	1	3	0	0	1	0	0	1.78
Standard deviation	1.05	0.97	1.10	0.95	1.17	1.10	1.02	1.07	0.85	0.78
Skewness (*SE* = 0.19)	0.02	− 0.44	0.05	− 0.75	0.07	0.31	0.24	0.38	1.77	− 0.03
Kurtosis (*SE* = 0.38)	− 1.18	− 0.79	− 1.31	− 0.47	− 1.48	− 1.27	− 1.06	− 1.10	2.25	− 0.92
Minimum	0	0	0	0	0	0	0	0	0	0.00
Maximum	3	3	3	3	3	3	3	3	3	3.00
Sample 3 (*N* = 372; Ukraine)										
Mean	0.87	0.79	0.79	1.11	0.60	0.52	0.66	0.69	0.17	0.69
Median	1.00	1.00	1.00	1.00	0.00	0.00	1.00	0.00	0.00	0.56
Mode	0	0	0	1	0	0	0	0	0	0.00
Standard deviation	0.88	0.85	0.94	0.90	0.89	0.74	0.77	0.83	0.50	0.62
Skewness (*SE* = 0.13)	0.83	0.81	0.99	0.50	1.35	1.44	1.03	1.07	3.24	1.07
Kurtosis (*SE* = 0.25)	− 0.02	− 0.18	0.00	− 0.49	0.75	1.70	0.61	0.46	11.30	0.57
Minimum	0	0	0	0	0	0	0	0	0	0.00
Maximum	3	3	3	3	3	3	3	3	3	2.67

The prevalence of occupational depression was 5% in Sample 1, 18% in Sample 2, and 3% in Sample 3. *SE* standard error, *ODI1* anhedonia, *ODI2* depressed mood, *ODI3* sleep alterations, *ODI4* fatigue/loss of energy, *ODI5* appetite alterations, *ODI6* feelings of worthlessness, *ODI7* cognitive impairment, *ODI8* psychomotor alterations, *ODI9* suicidal ideation.

#### Resilience scale

We used the Resilience Scale to assess resilience^28^. The Resilience Scale comprises 25 items rated from 0 for “definitely not” to 4 for “definitely.” The Resilience Scale covers various aspects of resilience, such as persistence in action, openness to novelty, reliance on humor, tolerance to failure, or optimism. Resilience items include: “I easily adapt to new situations”; “I consider myself a strong person”; “My life has meaning.” Cronbach’s α was 0.93. Only Sample 2 completed the Resilience Scale.

#### Areas of worklife scale

We assessed job-person fit using the Areas of Worklife Scale^20,29^. The instrument comprises six subscales assessing job-person fit in six domains: workload (5 items; Cronbach α = 0.77); control (4 items; Cronbach α = 0.83); rewards (4 items; Cronbach α = 0.88); community (5 items; Cronbach α = 0.81); fairness (6 items; Cronbach α = 0.83); and values (4 items; Cronbach α = 0.75). Each item was rated on a scale from 1 for “strongly disagree” to 5 for “strongly agree”. For each of the six domains, a higher score indicates a better fit. Only Sample 2 completed the Areas of Worklife Scale.

### Data analysis

**Figure 1:**
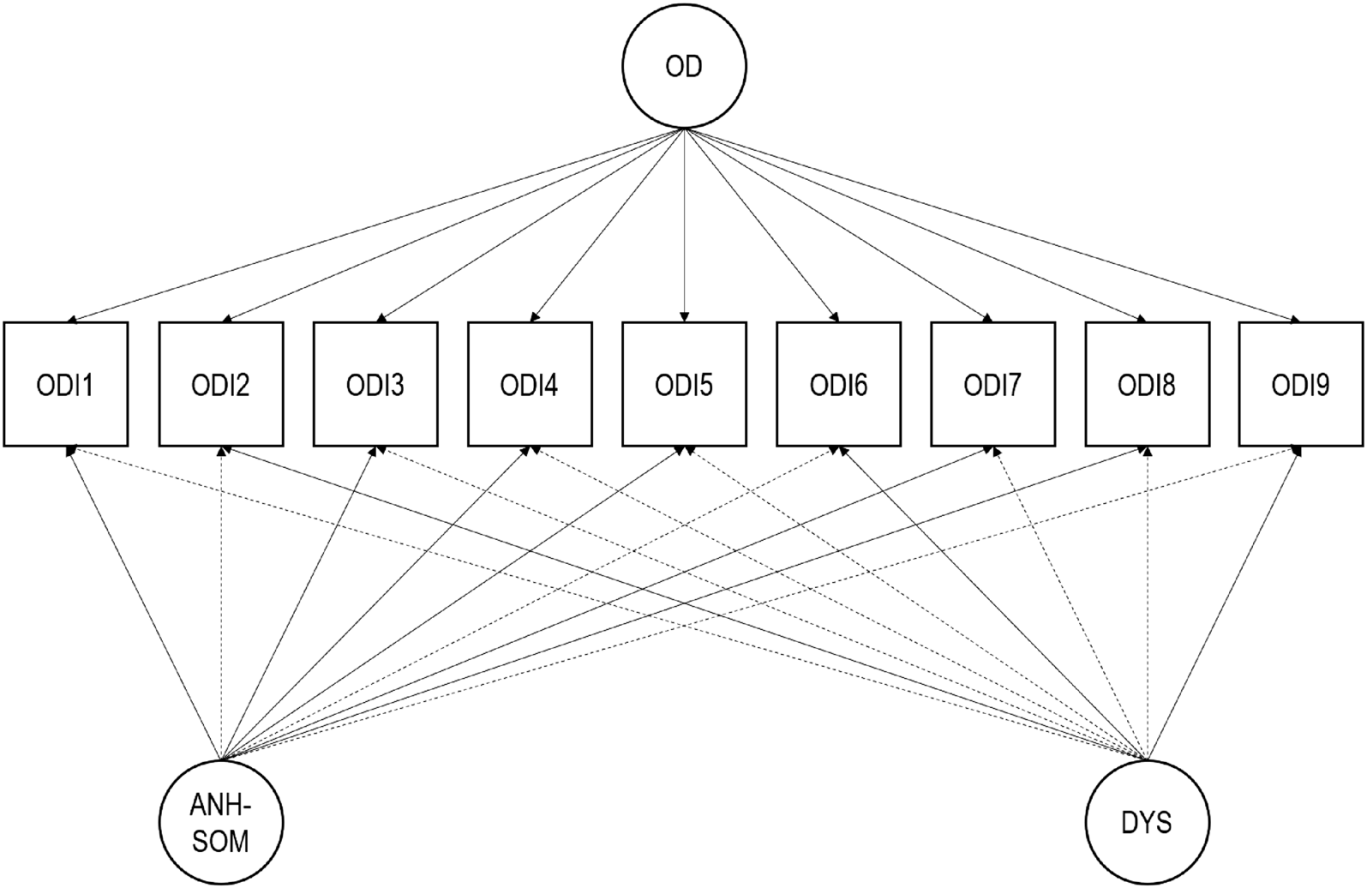
Exploratory structural equation modeling bifactor structure under examination. Solid lines are indicative of target loadings. Two specific factors are considered on account of the anhedonic-somatic and dysphoric symptom items populating the Occupational Depression Inventory. *OD* general Occupational Depression factor, *ANH-SOM* Anhedonic-Somatic specific factor, *DYS* Dysphoric specific factor, *ODI1* anhedonia, *ODI2* depressed mood, *ODI3* sleep alterations, *ODI4* fatigue/loss of energy, *ODI5* appetite alterations, *ODI6* feelings of worthlessness, *ODI7* cognitive impairment, *ODI8* psychomotor alterations, *ODI9* suicidal ideation.

Consistent with previous ODI studies, we examined the factorial structure of the ODI using ESEM bifactor analysis^30,31^. We considered two specific factors in addition to the general factor (Fig. 1), on account of the anhedonic-somatic and dysphoric symptom items populating the scale. In keeping with past research, we treated the items as ordinal, employed the weighted least squares—mean and variance adjusted—(WLSMV) estimator, and relied on a target rotation. We inspected the factor loadings on the general and specific factors and, as recommended, we computed the explained common variance (ECV) index to further estimate the importance of the general factor^31,32^. An ECV index exceeding 0.80 is suggestive of essential unidimensionality. The higher the ECV index, the greater the role of the general factor in accounting for the common variance extracted. We conducted our factor analyses in Mplus 8.7^33^.

We looked into the ODI’s homogeneity using the Mokken package version 3.0.6^34^ in R version 4.2.0^35^. Homogeneity refers to the extent to which a scale's items hierarchically align on a single dimension. The hierarchy concerns *item difficulty*, which, in this context, refers to the probability that an item will be endorsed by respondents. Homogeneity is indexed by *H* coefficients. As per commonly applied standards^36^, homogeneity is considered weak if 0.30 ≤ *H* < 0.40, moderate if 0.40 ≤ *H* < 0.50, and strong if *H* ≥ 0.50; a scale-level *H* coefficient below 0.30 suggests that the scale of interest cannot be regarded as unidimensional. Pairwise *H* coefficients should be > 0; item-level *H* coefficients should be > 0.30.

We investigated the total-score reliability of the ODI based on McDonald’s omega, Cronbach’s α, Guttman’s lambda-2, and the Molenaar-Sijtsma statistic. We relied on the Pearson product-moment correlation coefficient to examine the relationships between the ODI and our other measures of interest and inquire into the ODI’s criterion validity.

We examined the ODI’s cross-sample measurement invariance focusing on configural invariance (equivalence in factor structures), weak invariance (equivalence in factor loadings), strong invariance (equivalence in item thresholds), and strict invariance (equivalence in item residuals). The equivalence constraints are cumulative. We relied on common standards for detecting deviations from measurement invariance. Invariance violations were signaled by increases in the RMSEA exceeding 0.015 and decreases in the CFI and TLI exceeding 0.010^37,38^. In a first analysis, we scrutinized measurement invariance across our three samples. In a second analysis, we scrutinized measurement invariance across our three samples and the sample used in the original validation study of the ODI^5^. Involving original validation samples in subsequent validation studies is recommended during scale development^39,40^.

## Results

### Sample 1 (*N* = 526; Poland)

#### Factorial validity and dimensionality

The specified ESEM bifactor structure exhibited an acceptable fit: RMSEA = 0.04; CFI = 1.00; TLI = 1.00; SRMR = 0.01; *χ*^2^ (12) = 19.51. All ODI items loaded strongly on the general factor (*M* = 0.85, *SD* = 0.04). Items loadings on the bifactors were comparatively weak. The ECV index reached 0.91, meaning that the general factor accounted for 91% of the common variance extracted. Such a proportion indicates essential unidimensionality.

#### Homogeneity and reliability

The results of the homogeneity analysis are summarized in Table 3. The ODI demonstrated strong homogeneity. The scale-level *H* coefficient reached 0.68 with a standard error of only 0.02. All item-level *H* coefficients largely exceeded the 0.30 threshold, and none of the pairwise *H* coefficients were low. The most frequently endorsed item was Item 4 (fatigue/loss of energy), and the least frequently endorsed item was Item 9 (suicidal ideation). As can be seen from Table 3, the ODI exhibited high total-score reliability. McDonald’s omega, Cronbach’s α, Guttman’s lambda-2, and the Molenaar-Sijtsma statistic exceeded 0.90.

**Table 3:** Homogeneity analysis of the Occupational Depression Inventory.

Items	Sample 1 (*N* = 526 [Poland])			Sample 2 (*N* = 164 [Poland])			Sample 3 (*N* = 372 [Ukraine])		
	*H*_*i*_	*SE*	95% CI	*H*_*i*_	*SE*	95% CI	*H*_*i*_	*SE*	95% CI
ODI1 (anhedonia)	0.69	0.02	[0.65, 0.73]	0.62	0.04	[0.55, 0.69]	0.62	0.03	[0.57, 0.68]
ODI2 (depressed mood)	0.70	0.02	[0.65, 0.74]	0.60	0.05	[0.51, 0.69]	0.66	0.02	[0.61, 0.70]
ODI3 (sleep alterations)	0.70	0.02	[0.66, 0.74]	0.57	0.04	[0.49, 0.65]	0.61	0.03	[0.55, 0.66]
ODI4 (fatigue/loss of energy)	0.65	0.03	[0.59, 0.70]	0.64	0.04	[0.57, 0.72]	0.64	0.03	[0.58, 0.70]
ODI5 (appetite alterations)	0.67	0.02	[0.63, 0.72]	0.60	0.04	[0.53, 0.68]	0.57	0.03	[0.50, 0.63]
ODI6 (feelings of worthlessness)	0.67	0.02	[0.62, 0.72]	0.50	0.05	[0.40, 0.59]	0.53	0.04	[0.45, 0.60]
ODI7 (cognitive impairment)	0.72	0.02	[0.67, 0.76]	0.58	0.04	[0.50, 0.66]	0.60	0.03	[0.54, 0.66]
ODI8 (psychomotor alterations)	0.69	0.02	[0.64, 0.73]	0.59	0.04	[0.50, 0.67]	0.62	0.03	[0.57, 0.68]
ODI9 (suicidal ideation)	0.66	0.03	[0.61, 0.72]	0.60	0.05	[0.50, 0.70]	0.51	0.05	[0.41, 0.61]
*H*	0.68	0.02	[0.65, 0.72]	0.59	0.03	[0.52, 0.65]	0.60	0.02	[0.55, 0.65]
McDonald’s omega	0.94			0.91			0.91		
Cronbach’s α	0.94			0.90			0.90		
Guttman’s lambda-2	0.94			0.91			0.91		
Molenaar-Sijtsma statistic	0.94			0.91			0.91		

*H* scale-level *H*, *H*_*i*_ item-level *H*, *SE* standard error, *CI* confidence interval. None of the pairwise *H* coefficients were low (≥ 0.54 in Sample 1; ≥ 0.42 in Sample 2; ≥ 0.32 in Sample 3).

### Sample 2 (*N* = 164; Poland)

#### Factorial validity and dimensionality

The specified ESEM bifactor structure represented an over-factored solution in Sample 2. We thus switched to a fully unidimensional confirmatory factor analytic model—all ODI items were allowed to load on a single factor with no secondary dimensions involved. The model showed an acceptable fit: RMSEA = 0.08; CFI = 0.99; TLI = 0.98; SRMR = 0.06; *χ*^2^ (27) = 57.44. Factor loadings ranged from 0.67 to 0.86 (*M* = 0.79, *SD* = 0.06).

#### Homogeneity and reliability

The homogeneity of the ODI was strong (scale-level *H* = 0.59, standard error = 0.03), with no problematic *H* values at either item or pairwise levels (Table 3). Again, the least difficult item was Item 4 (fatigue/loss of energy), and the most difficult item was Item 9 (suicidal ideation). As was the case in Sample 1, the ODI showed high total-score reliability. McDonald’s omega, Cronbach’s α, Guttman’s lambda-2, and the Molenaar-Sijtsma statistic were ≥ 0.90 (see Table 3).

#### Criterion validity

Occupational depression correlated, in the expected direction, with resilience and job-person fit in six areas of working life—workload, control, rewards, community, fairness, and values (Table 4). Moderate to large correlations were observed. Absolute *r*s ranged from 0.24 (for values) to 0.49 (for workload).

**Table 4:** Criterion validity analysis (Sample 2 [Poland]).

Potential correlate of occupational depression	*M*	*SD*	*r*	*p*	95% CI*	
Resilience	2.35	0.68	− 0.29	< 0.001	− 0.43	− 0.13
Job-person fit—workload	2.45	0.85	− 0.49	< 0.001	0.36	0.59
Job-person fit—control	3.37	0.96	− 0.35	< 0.001	− 0.48	− 0.21
Job-person fit—rewards	3.12	0.99	− 0.34	< 0.001	− 0.47	− 0.20
Job-person fit—community	3.21	0.82	− 0.27	< 0.001	− 0.41	− 0.12
Job-person fit—fairness	2.71	0.88	− 0.30	< 0.001	− 0.44	− 0.16
Job-person fit—values	3.02	0.83	− 0.24	0.002	− 0.38	− 0.09

*Estimation is based on Fisher's *r*-to-*z* transformation with bias adjustment. *N*s vary from 140 to 164 due to missing values; there were no missing values for occupational depression (or any of the items of the Occupational Depression Inventory). *CI* confidence interval.

### Sample 3 (*N* = 372; Ukraine)

#### Factorial validity and dimensionality

The specified ESEM bifactor structure exhibited an acceptable fit: RMSEA = 0.00; CFI = 1.00; TLI = 1.00; SRMR = 0.01; *χ*^2^ (12) = 11.29. All ODI items loaded strongly on the general factor (*M* = 0.78, *SD* = 0.08). Items loadings on the bifactors were comparatively weak. The ECV index reached 0.85, a value suggestive of essential unidimensionality.

#### Homogeneity and reliability

The results of the homogeneity analysis are summarized in Table 3. The ODI demonstrated strong homogeneity. The scale-level *H* coefficient reached 0.60 with a standard error of only 0.02. All item-level *H* coefficients largely exceeded the 0.30 threshold, and none of the pairwise *H* coefficients were low. The most frequently endorsed item was Item 4 (fatigue/loss of energy), and the least frequently endorsed item was Item 9 (suicidal ideation). As can be seen from Table 3, the ODI exhibited high total-score reliability. McDonald’s omega, Cronbach’s α, Guttman’s lambda-2, and the Molenaar-Sijtsma statistic ≥ 0.90.

### Measurement invariance

As can be seen from Supplemental Material 3, we found measurement invariance to hold across our three samples considered separately as well as across our three samples and the original validation sample of the ODI. As we added constraints from configural invariance to strict invariance, none of the fit indices showed problematic alterations. As an illustration, CFI never decreased by more than 0.002, and TLI never decreased by more than 0.003. Complete invariance was thus reached.

## Discussion

The ODI reflects a renewed approach to job-related distress and is increasingly used across countries and linguistic communities. The present study inquired into the psychometric and structural properties of the ODI’s Polish and Ukrainian versions. Such an inquiry is important to ascertain whether Polish- and Ukrainian-speaking occupational health specialists can confidently use the instrument. We relied on up-to-date statistical techniques to accomplish our research goals.

### Main findings

In all samples, the ODI met the requirements for essential unidimensionality. These results are consistent with the findings obtained in all previous ODI studies^5,6,11–13,16,17^. Homogeneity analysis indicated that the items of the ODI hierarchically align on a single dimension, in keeping with ODI studies conducted in other geographic and linguistic contexts^6,11–13,16,17^. These findings suggest that the ODI’s total score accurately ranks respondents on the latent continuum underlying the scale.

We found the ODI to exhibit strong total-score reliability. This finding was documented based on four different indicators. It is of note that the ODI displays strong total-score reliability despite covering nine symptoms and showing no explicit redundancy in the content of its items. It is well-known that, in many scales, total-score reliability (typically indexed by Cronbach’s α) is inflated by the repetition of nearly identical, virtually interchangeable questions^41,42^. The ODI’s total-score reliability is unlikely to be biased upward by item synonymy because each of the ODI’s items covers a specific symptom of major depression. More probably, the unity of the item set reflects clinically meaningful bonds among the symptoms assessed.

Speaking to the criterion validity of the ODI, we found occupational depression to correlate negatively with resilience and job-person fit in six areas of working life—workload, control, rewards, community, fairness, and values. The present study is the first to examine the nomological network of occupational depression in relation to these variables. Our results are consistent with the findings pertaining to general depressive symptoms and disorders. Depression has been linked to a lack of resilience in past research^43^. Leiter and Maslach (2004) reported small to large correlations between workload, control, rewards, community, fairness, and values as operationalized by the Areas of Worklife Scale and *burnout*—an entity known to overlap with (occupational) depression^7,44,45^.

The availability of uncomplicated, yet dependable, assessment tools is of paramount importance in identifying deteriorated health and impaired functioning in the workplace. The Polish and Ukrainian versions of the ODI can serve as both a research asset and a signaler to take preventive and interventional measures in organizations.

### Study limitations and strengths

The present study has at least three limitations. First, we relied on a cross-sectional design, which prevented us from addressing properties such as test–retest reliability. Second, the alpha reliabilities of some of the subscales of the Areas of Worklife Scale were in the 0.70s, a range that is considered barely adequate in the context of basic research^46^. Our alpha reliabilities, however, comport with those documented by the creators of the Areas of Worklife Scale^20^. Third, we did not inquire into the ODI’s discriminant validity vis-à-vis attribution-free measures of depression. Fortunately, the ODI’s discriminant validity has been examined in several past ODI studies based on a variety of depression scales^5,16,17^.

As for its strengths, the present study involved three different samples, thus incorporating a replication component within its design. In addition, the study relied on advanced statistical techniques attached to classical test theory and item response theory. The techniques employed allowed us to conduct both synoptic and granular analyses of the data. Combined with the use of three different samples, this modus operandi increases the conclusiveness of the study findings. That the ODI showed complete measurement invariance across our samples suggests that differences in observed scores reflect genuine symptom variations rather than artifacts related to idiosyncratic utilization of the scale. Reaching complete measurement invariance is highly important for a measure. Strict invariance is indeed a prerequisite for between-group comparisons involving observed scores^37,38^.

We note that the Ukrainian data were collected in April 2023, i.e., in war times. It is likely that the war context influenced workers and organizations in the country. The nature of that influence on occupational depression is, however, unclear. On the one hand, the war context and its tragedies may have produced a “relativism effect” leading individuals to be less sensitive to work-related hassles and stressors. On the other hand, at least in some occupations, job demands may have increased dramatically, and job resources may have decreased considerably, setting a perfect floor for job strain and mental health issues. Unfortunately, our study does not allow us to clarify such questions. While it is tempting to call for further research, conducting further research is challenging in the current circumstances.

### Conclusions

The Polish and Ukrainian versions of the ODI exhibit robust psychometric and structural properties. Given the estimated health and economic cost of job-related distress, it is crucial for occupational health specialists to assess the phenomenon reliably and validly. In view of its characteristics, and because it is available free of charge, the ODI has the potential to help researchers, practitioners, and public health decision-makers address job-related distress effectively.

### Supplementary Information

Supplementary Information 1.

Supplementary Information 2.

Supplementary Information 3.

## Data Availability

The datasets used in the current study are available from the corresponding author upon reasonable request.
