# Supplementary material for: A validation study of the Occupational Depression Inventory in Poland and Ukraine
Source: Sci Rep. 2024 Feb 22;14:4403. doi: 10.1038/s41598-024-54995-w (PMC10883996; doi:10.1038/s41598-024-54995-w)
Supplement: Supplementary file 1 — Supplementary Information 1. [file 41598_2024_54995_MOESM1_ESM.pdf]

Occupational Depression Inventory: SPSS syntax for a provisional diagnosis of occupational depression (POLAND)

```
compute DEP = 0.
do if ODI1 ge 3 or ODI2 ge 3.
count DEP = ODI3 (3)
ODI4 (3)
ODI5 (3)
ODI6 (3)
ODI7 (3)
ODI8 (3)
ODI9 (1,2,3).
end if.

if ODI1 ge 3 DEP = DEP + 1.
if ODI2 ge 3 DEP = DEP + 1.

compute DIAG = 0.
if DEP ge 5 DIAG = 1.
```

Note. The nine items of the Occupational Depression Inventory are coded ODI1 to ODI9.

Items

ODI1: anhedonia

ODI2: depressed mood

ODI3: sleep alterations

ODI4: fatigue/loss of energy

ODI5: appetite alterations

ODI6: feelings of worthlessness

ODI7: cognitive impairment

ODI8: psychomotor alterations

ODI9: suicidal ideation

The Polish version of the scale is displayed right below.

↓↓↓↓↓

INWENTARZ DEPRESJI ZAWODOWEJ (IDZ)

WSTĘPNE INSTRUKCJE DLA RESPONDENTÓW

Poniższe stwierdzenia dotyczą wpływu, jaki mogła mieć na Ciebie praca.

Odnosząc się do każdego stwierdzenia oceń, jak często doświadczałeś/aś wymienionych problemów w ciągu OSTATNICH 2 TYGODNI. Aby odpowiedzieć, użyj następującej skali:

0 = nigdy lub prawie nigdy

1 = tylko przez kilka dni

2 = więcej niż przez połowę dni

3 = prawie codziennie

Przykład:

„Odczuwałem/am niepokój z powodu mojej pracy."

- Jeśli NIE odczuwałeś/aś niepokoju z powodu swojej pracy, zaznacz **0.**
- Jeśli odczuwałeś/aś niepokój z powodów NIEZWIĄZANYCH Z PRACĄ (problemy osobiste, małżeńskie, rodzinne, zdrowotne itp.), również zaznacz **0.**
- Jeśli odczuwałeś/aś niepokój, ale nie wiesz dlaczego, także zaznacz **0.**
- Jeśli wiesz, że to TWOJA PRACA wywoływała niepokój, zaznacz **1**, **2** lub **3**, aby wskazać, jak często się to zdarzało.

Teraz możesz wypełnić kwestionariusz.

INWENTARZ DEPRESJI ZAWODOWEJ (IDZ)

Dane Pacjenta: ………………………………………………………………… Data: …………………………………………

Oceń, jak często doświadczałeś/aś wymienionych problemów w ciągu ostatnich 2 tygodni.	*Nigdy lub prawie nigdy*	*Tylko przez kilka dni*	*Więcej niż przez połowę dni*	*Prawie codziennie*
1. Moja praca była tak stresująca, że nie mogłem/am cieszyć się rzeczami, które zwykle lubię robić.	**0**	**1**	**2**	**3**
2. Czułem/am się przygnębiony/a z powodu mojej pracy.	**0**	**1**	**2**	**3**
3. Stres związany z pracą spowodował, że miałem/am problemy ze snem (miałem/am trudności z zasypianiem, wybudzałem/am się lub spałem/am znacznie więcej niż zwykle).	**0**	**1**	**2**	**3**
4. Czułem/am się wyczerpany/a swoją pracą.	**0**	**1**	**2**	**3**
5. Czułem/am, że stres w pracy wpłynął na mój apetyt (straciłem/am apetyt lub przeciwnie, jadłem/am za dużo).	**0**	**1**	**2**	**3**
6. Moje doświadczenie w pracy sprawiają, że czuję się jak nieudacznik.	**0**	**1**	**2**	**3**
7. Moja praca tak bardzo mnie stresowała, że miałem/am problem ze skupieniem się na wykonywanych czynnościach (np. na czytaniu artykułu w gazecie) lub z jasnym myśleniem (np. podejmowaniem decyzji).	**0**	**1**	**2**	**3**
8. W wyniku stresu w pracy czułem/am się niespokojny/a lub wręcz przeciwnie, czułem/am wyraźne spowolnienie - np. w sposobie poruszania się lub mówienia.	**0**	**1**	**2**	**3**
9. Pomyślałem/am, że wolałbym/abym umrzeć niż pozostać w tej pracy.	**0**	**1**	**2**	**3**

WYNIK OGÓŁEM: ………………………………………………………

Jeśli napotkałeś przynajmniej niektóre z wyżej wymienionych problemów, czy te problemy sprawiają, że zastanawiasz się nad odejściem z obecnej pracy lub stanowiska?

☐ Tak ☐ Nie ☐ Nie wiem
