# Supplementary material for: A validation study of the Occupational Depression Inventory in Poland and Ukraine
Source: Sci Rep. 2024 Feb 22;14:4403. doi: 10.1038/s41598-024-54995-w (PMC10883996; doi:10.1038/s41598-024-54995-w)
Supplement: Supplementary file 2 — Supplementary Information 2. [file 41598_2024_54995_MOESM2_ESM.pdf]

Occupational Depression Inventory: SPSS syntax for a provisional diagnosis of occupational depression (UKRAINE)

```
compute DEP = 0.
do if ODI1 ge 3 or ODI2 ge 3.
count DEP = ODI3 (3)
ODI4 (3)
ODI5 (3)
ODI6 (3)
ODI7 (3)
ODI8 (3)
ODI9 (1,2,3).
end if.

if ODI1 ge 3 DEP = DEP + 1.
if ODI2 ge 3 DEP = DEP + 1.

compute DIAG = 0.
if DEP ge 5 DIAG = 1.
```

Note. The nine items of the Occupational Depression Inventory are coded ODI1 to ODI9.

Items

ODI1: anhedonia

ODI2: depressed mood

ODI3: sleep alterations

ODI4: fatigue/loss of energy

ODI5: appetite alterations

ODI6: feelings of worthlessness

ODI7: cognitive impairment

ODI8: psychomotor alterations

ODI9: suicidal ideation

The Ukrainian version of the scale is displayed right below.

↓↓↓↓↓

ШКАЛА ДЕПРЕСІЇ, ПОВ’ЯЗАНОЇ З РОБОТОЮ

OCCUPATIONAL DEPRESSION INVENTORY (ODI)

ПОПЕРЕДНІ ІНСТРУКЦІЇ ДЛЯ РЕСПОНДЕНТІВ

Наведені нижче твердження стосуються того, як на Вас впливає Ваша робота.

Будь ласка, прочитайте кожне твердження і вкажіть, як часто Ви відчували такі проблеми за ОСТАННІ ДВА ТИЖНІ. Використовуйте надану шкалу для відповіді:

0 = ніколи або майже ніколи

1 = лише кілька днів

2 = більшість днів

3 = майже кожен день

Ось приклад:

“Я відчував(ла) тривогу через свою роботу.”

• Якщо Ви НЕ відчували тривогу через свою роботу, виберіть 0.

• Якщо Ви відчували тривогу з причин, які Ви вважаєте НЕПОВ'ЯЗАНИМИ З ВАШОЮ РОБОТОЮ (особисті проблеми, проблеми у шлюбі, сімейні проблеми, проблеми зі здоров'ям тощо), також виберіть 0.

• Якщо ви відчували тривогу, але не знаєте, чому, знову виберіть 0.

• Якщо вам зрозуміло, що ВАША РОБОТА спричинила вам тривогу, виберіть 1, 2 або 3, щоб показати, як часто це відбувалося.

Зараз Ви можете заповнити анкету.

ШКАЛА ДЕПРЕСІЇ, ПОВ'ЯЗАНОЇ З РОБОТОЮ

Ім'я: ………………………………………………………………………… Дата: …………………………………………

**Вкажіть, з якою частотою Ви відчували наведені нижче проблеми протягом останніх двох тижнів.**	*ніколи або майже ніколи*	*лише кілька днів*	*більшість днів*	*майже кожен день*
1. Моя робота була настільки напруженою, що я не міг(могла) насолоджуватись тим, що зазвичай люблю робити.	**0**	**1**	**2**	**3**
2. Я почував(ла) себе пригніченим(ною) через мою роботу.	**0**	**1**	**2**	**3**
3. Стрес на роботі призвів до проблем зі сном (мені було важко заснути або підтримувати сон, або я спав(ла) набагато більше, ніж зазвичай).	**0**	**1**	**2**	**3**
4. Я почував(ла) себе виснаженим(ною) через мою роботу.	**0**	**1**	**2**	**3**
5. Я відчував(ла), що мій апетит порушився через стрес на роботі (я втратив(ла) апетит, або, навпаки, їв(їла) занадто багато).	**0**	**1**	**2**	**3**
6. Те, що я переживав(ла) на роботі, змусило мене відчувати себе невдахою.	**0**	**1**	**2**	**3**
7. Моя робота дуже мене напружувала, і я мав(ла) проблеми з концентрацією на тому, що роблю (наприклад, читання статті), або з ясним мисленням (наприклад, прийняття рішення).	**0**	**1**	**2**	**3**
8. Унаслідок стресу на роботі я відчував(ла) себе неспокійним(ною), або, навпаки, помітно уповільненим(ною) - наприклад, це проявлялось у тому, як я рухався(лась) або говорив(ла).	**0**	**1**	**2**	**3**
9. Я думав(ла), що краще вмерти, ніж продовжувати працювати на цій роботі.	**0**	**1**	**2**	**3**

ЗАГАЛЬНИЙ БАЛ: ………………………………………………………

Якщо ви стикнулися хоча б з деякими з проблем, зазначених вище, чи ці проблеми привели вас до розгляду питання про звільнення з вашої поточної роботи або посади?

☐ Так ☐ Ні ☐ Я не знаю
