# Supplementary material for: A validation study of the Occupational Depression Inventory in Poland and Ukraine
Source: Sci Rep. 2024 Feb 22;14:4403. doi: 10.1038/s41598-024-54995-w (PMC10883996; doi:10.1038/s41598-024-54995-w)
Supplement: Supplementary file 3 — Supplementary Information 3. [file 41598_2024_54995_MOESM3_ESM.pdf]

Supplemental Material 3. Measurement invariance across samples.

Invariance model	χ^2 (df)	CFI	TLI	RMSEA [90% CI]	CM	$\Delta\chi^2$	Δdf	ΔCFI	ΔTLI	ΔRMSEA
Three-sample analysis										
1. Configural	51.829 (36)	0.999	0.998	0.035 [0.007, 0.055]	—	—	—	—	—	—
2. Weak (λ)	133.497 (72)	0.997	0.995	0.049 [0.036, 0.062]	1	81.488*	36	-0.002	-0.003	0.014
3. Strong (λ, τ)	211.080 (102)	0.995	0.994	0.055 [0.044, 0.065]	2	79.893*	30	-0.002	-0.001	0.006
4. Strict (λ, τ, δ)	244.344 (120)	0.994	0.994	0.054 [0.044. 0.064]	3	42.380*	18	-0.001	0.000	-0.001
Four-sample analysis										
1. Configural	117.835 (48)	0.999	0.997	0.042 [0.032, 0.052]	—	—	—	—	—	—
2. Weak (λ)	278.536 (102)	0.997	0.996	0.046 [0.039, 0.052]	1	150.238*	54	-0.002	-0.001	0.004
3. Strong (λ, τ)	396.475 (147)	0.996	0.996	0.045 [0.040, 0.051]	2	133.630*	45	-0.001	0.000	-0.001
4. Strict (λ, τ, δ)	467.018 (174)	0.995	0.996	0.045 [0.040, 0.050]	3	84.607*	27	-0.001	0.000	0.000

Notes. * $p < .01$; $\Delta\chi^2$ chi-square difference test calculated using the Mplus DIFFTEST option (reported for descriptive purposes); CM = comparison model; CI = confidence interval; λ = factor loadings; τ = thresholds; δ = uniquenesses. The three-sample analysis involves our three study samples (i.e., our two Polish samples and our Ukrainian sample). The four-sample analysis additionally involves the 2254-participant sample used in the inaugural validation study of the Occupational Depression Inventory.
